# Eco-friendly spectrofluorimetric determination of fenbendazole in pharmaceutical, plasma, and milk samples using erythrosine B as a fluorescent probe

**DOI:** 10.1039/d6ra04629k

**Published:** 2026-09-29

**Authors:** Ahmad A. A. Mohamad, Ahmed A. Almrasy, Ahmed H. Abdelazim, Ahmed S. Taha

**Affiliations:** a Pharmaceutical Analytical Chemistry Department, Faculty of Pharmacy, Al-Azhar University Cairo 11751 Egypt ahmedsobhytaha@gmail.com

## Abstract

A simple, sensitive, and sustainable spectrofluorimetric method was developed to determine fenbendazole (FEN) based on its fluorescence-quenching interaction with erythrosine B (EB). Under the optimized conditions, FEN efficiently quenched the native fluorescence of EB (*λ*_ex_ = 527 nm, *λ*_em_ = 544 nm). The decrease in fluorescence intensity was proportional to FEN concentration over 0.050–3.0 µg mL^−1^ (*r*^2^ = 0.9997), with limits of detection and quantification of 0.014 and 0.043 µg mL^−1^, respectively. Stern–Volmer analysis, thermodynamic studies, Job's method, spectral analysis, and quantum-chemical calculations collectively supported a predominantly static quenching mechanism involving a spontaneous 1 : 1 ground-state EB–FEN complex, with electrostatic interactions, hydrogen bonding, and partial charge transfer contributing to the interaction. The method was validated according to ICH Q_2_(R2) guidelines and showed satisfactory accuracy, precision, robustness, and selectivity for FEN determination in pharmaceutical formulations and spiked bovine plasma and milk samples following sample pretreatment. The sustainability of the proposed method was evaluated using AGREEprep, ComplexMoGAPI, CaFRI, BAGI, and RGB12, showing favorable environmental performance while acknowledging the acetonitrile used during plasma pretreatment. Because of its simplicity, speed, low solvent consumption, and modest instrumental requirements, the proposed method offers a practical complement to chromatographic methods for routine FEN determination in pharmaceutical formulations and spiked biological matrices.

## Abstract 2

A simple, sensitive, and sustainable spectrofluorimetric method was developed to determine fenbendazole (FEN) based on its fluorescence-quenching interaction with erythrosine B (EB).

## Graphical abstract

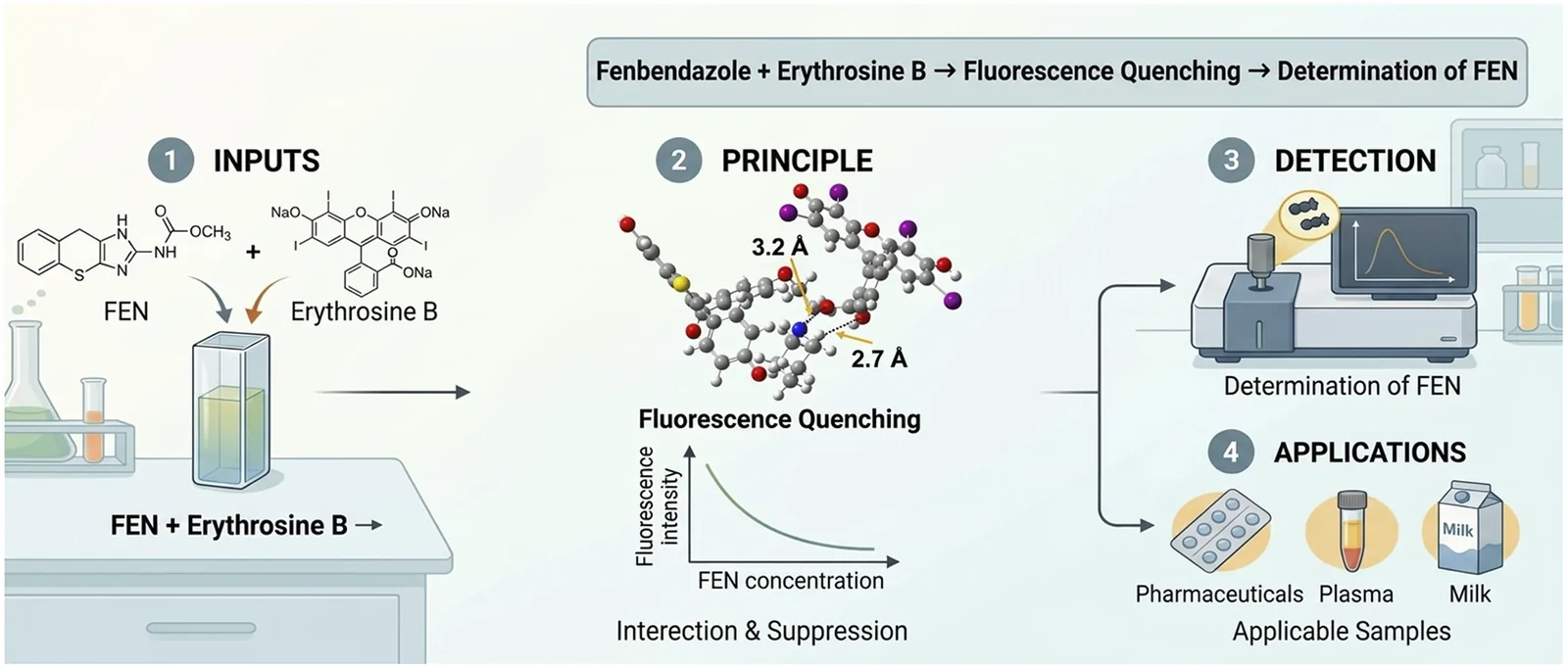

## 1 Introduction

Fenbendazole (FEN) is a broad-spectrum benzimidazole anthelmintic widely used in veterinary medicine to control and treat gastrointestinal helminth infections in food-producing and companion animals.^1,2^ Its an3helmintic effect is attributed to selective binding to parasite β-tubulin, which inhibits microtubule polymerization, disrupts glucose uptake, and ultimately causes parasite death.^3,4^ Owing to its established efficacy and favorable safety profile, FEN remains an important veterinary drug in livestock and companion animal practice. In addition, FEN has recently attracted growing scientific interest due to reports of potential anticancer activity.^5–7^ These developments have further increased the demand for reliable analytical methods to determine FEN in pharmaceutical formulations and biological matrices relevant to veterinary analysis. From an analytical perspective, FEN is poorly soluble in water and has limited oral bioavailability, which may complicate sample preparation and quantitative analysis in complex matrices.^8,9^ These characteristics highlight the need for reliable analytical methods for pharmaceutical quality control and biological matrix analysis. At the same time, matrix constituents present in biological samples may complicate quantitative analysis, making appropriate sample preparation and method validation essential. A variety of analytical methods have been reported for FEN determination, including chromatographic, spectrophotometric, and electrochemical methods.^10–18^ High-performance liquid chromatography (HPLC) and LC-MS/MS provide excellent analytical performance; however, they generally require sophisticated instrumentation and, in many applications, relatively more demanding sample preparation procedures. Therefore, there is continued interest in developing simpler, faster, and more environmentally benign alternatives suitable for routine analysis.^19,20^ Spectrofluorimetric methods are attractive in this context because they combine high sensitivity with simplicity, rapidity, and low reagent consumption.^21–25^ In particular, fluorescent dyes such as erythrosine B (EB) have been successfully used as probes to determine several drugs *via* fluorescence quenching or complex formation.^26–29^ This analytical strategy provides a convenient platform for method development; however, its performance depends strongly on the physicochemical interaction between the probe and the analyte, as well as on the sample matrix composition. For this reason, careful optimization and validation are essential when applying such methods to pharmaceutical and biological samples.^30–34^ To the best of our knowledge, no spectrofluorimetric method based on EB has been reported for determining FEN in pharmaceutical formulations and in fortified bovine plasma and milk samples. Accordingly, the present work aimed to develop and validate a simple, sensitive, and environmentally sustainable turn-off spectrofluorimetric method for determining FEN based on its interaction with EB. The proposed method was optimized and validated according to ICH Q2(R2) guidelines for linearity, sensitivity, selectivity, accuracy, precision, and robustness.^35^ Stern–Volmer analysis, thermodynamic studies, Job's method, spectral analysis, and quantum-chemical calculations were employed to gain molecular-level insight into the FEN–EB interaction and the proposed quenching mechanism. The environmental sustainability and overall analytical performance were further evaluated using AGREEprep, CaFRI, ComplexMoGAPI, BAGI, and RGB12.^36–40^

## 2 Experimental

### 2.1 Materials and reagents

FEN standard (99.8% purity) was obtained from ATCO Pharma for Pharmaceuticals Industries (El Obour City, Egypt). Pharmaceutical tablets containing FEN (Panacur 150 mg Vet tablets®) were purchased from local pharmacies (Cairo, Egypt). Erythrosine B dye (95% dye content) and HPLC-grade ethanol were procured from Sigma-Aldrich (St. Louis, MO, USA). Distilled water was used to prepare all solutions. EB was prepared as a 0.02% w/v stock solution in distilled water analysis. Blood samples were collected into heparinized tubes and centrifuged at 4000 rpm for 10 min to separate plasma, which was then stored at −20 °C until use.

Fresh milk samples were collected from healthy cattle at a local dairy farm in Al Sharkia, Egypt, and stored at −20 °C until analysis. Before use, samples were thawed at room temperature and homogenized.

Britton–Robinson universal buffer solutions were freshly made before each analytical session in compliance with USP standards.

### 2.2 Instrumentation

For spectrophotometric measurements, a Shimadzu UV-1800 double-beam UV-vis spectrophotometer was used with matching 1 cm quartz cells. The fluorescence measurements were taken with a Jasco FP-6200 spectrofluorometer equipped with 1.0 cm quartz cells and a 150 W xenon lamp. After this optimization, the parameters were fine-tuned to an excitation wavelength of 527 nm and an emission wavelength of 544 nm. To facilitate excitation and emission, slit widths of 10 nm were instituted. Utilizing Spectra Manager 2.4 software, data collection and spectral processing were overseen. A calibrated Jenway 3510 pH meter (Staffordshire, UK) was used to measure pH. Computational calculations were carried out using the software package Gaussian 09 (Gaussian Inc., Wallingford, CT, USA).

### 2.3 Standard solutions and fluorescent probe construction

A stock solution of FEN (100 µg mL^−1^) was prepared by dissolving 10 mg of FEN in 10 mL of ethanol. The final stock solution was prepared by diluting it to 100 mL with distilled water. A working standard solution (20 µg mL^−1^) was prepared by transferring 20 mL of the stock solution into a 100 mL volumetric flask and diluting to volume with a binary solvent system of ethanol and distilled water (1 : 10, v/v). To prepare the fluorescent probe, accurately transfer 2.0 mL of EB solution (0.01% w/v) and 2.0 mL of Britton–Robinson buffer (pH 3.0) into a 10 mL volumetric flask and dilute to the mark with distilled water. The fluorescence measurements were carried out at an excitation wavelength (*λ*_ex_) of 527 nm and an emission wavelength (*λ*_em_) of 554 nm. The initial fluorescence intensity (*F*_0_) of this probe solution was measured before the addition of the analyte.

Aliquots of the working FEN solution (20 µg mL^−1^) were transferred into a series of 10 ml volumetric flasks to give final concentrations in the range of 0.05–2.0 µg mL^−1^ each flask was added with 2.0 mL of EB solution (0.01% w/v), giving a final dye concentration of 20.0 µg mL^−1^ and 2.0 mL of Britton–Robinson buffer (pH 3.0). The solutions were mixed thoroughly and diluted to volume with distilled water. The fluorescence intensity (*F*) of the solutions was measured under optimized conditions after a 3 min incubation at room temperature. The quenching behavior was analyzed by plotting the Stern–Volmer relationship (*F*_0_/*F*) *versus* FEN concentration to obtain the calibration curve. This linear relationship was then used to quantitatively determine FEN.

### 2.4 Studies on mechanistic investigation

#### 2.4.1 Stern–Volmer analysis

Stern–Volmer analysis was performed at three temperatures (298, 303, and 313 K) using a constant concentration of EB (20 µg mL^−1^) and FEN concentrations ranging from 0.5 to 3.0 µg mL^−1^. We added appropriate aliquots of the FEN working solution to obtain the required final concentrations. The solutions were buffered at pH 3.0, and fluorescence intensities were recorded after 3 min of equilibration at each investigated temperature.

The fluorescence intensity ratio (*F*_0_/*F*) was plotted against the molar concentration of FEN ([*Q*]), and the Stern–Volmer equation was applied: *F*_0_/*F* = 1 + *K*_sv_[*Q*].where *F*_0_ and *F* are the fluorescence intensities of EB in the absence and presence of FEN, respectively; *K*_sv_ is the Stern–Volmer quenching constant; and [*Q*] is the molar concentration of FEN. We evaluated the effect of temperature on the obtained *K*_sv_ values to characterize the predominant quenching mechanism. For binding analysis, the association constant was additionally evaluated using:*F*_0_/*F*_0_ − *F* = 1 + 1/*K*_a_[*Q*]where *K*_a_ represents the apparent association constant. The logarithmic binding equation was also applied as a complementary approach to estimate the apparent number of binding sites:Log(*F*_0_ − *F*/*F*) = log *K*_a_ + *n *log[*Q*]where *n* represents the apparent number of binding sites; the resulting binding parameters were evaluated in conjunction with the stoichiometric information obtained from Job's method.^25,27–42^

#### 2.4.2 Thermodynamic investigations

The thermodynamic parameters, including the changes in Gibbs free energy (Δ*G*), enthalpy (Δ*H*), and entropy (Δ*S*), were determined from the temperature-dependent binding studies conducted at 298, 303, and 313 K. The association constant (*K*_a_) at each temperature was obtained using the modified Stern–Volmer relationship:*F*_0_/(*F*_0_ − *F*) = 1 + 1/(*K*_a_[*Q*])where *F*_0_ and *F* are the fluorescence intensities in the absence and presence of FEN, respectively, and [*Q*] is the molar concentration of FEN. The van't Hoff equation was used to determine Δ*H* and Δ*S*:Ln *K*_a_ = −Δ*H*/(*RT*) + Δ*S*/*R*where *R* is the universal gas constant, and *T* is the absolute temperature. The Gibbs free energy change was subsequently calculated using:Δ*G* = Δ*H* − *T*Δ*S*.

The temperature-dependent association constants and the calculated thermodynamic parameters were used to evaluate the spontaneity and thermal behavior of the EB–FEN association.^43,44^

#### 2.4.3 Job's method for stoichiometric analysis

The stoichiometric ratio of the EB–FEN association complex was investigated using Job's method of continuous variation. Under the optimized experimental conditions, we prepared a series of solutions at a constant total molar concentration of 1.0 × 10^−5^ M. In contrast, the molar fractions of EB and FEN were systematically varied over the range of 0.1–0.9. The fluorescence response was expressed as Δ*F* (*F*_0_ − *F*), and Δ*F* was plotted against the mole fraction of FEN. The stoichiometric ratio was determined from the mole fraction corresponding to the maximum response in the Job's plot.^45,46^

#### 2.4.4 Density functional theory (DFT) calculations

Density functional theory (DFT) calculations were performed using Gaussian 09 to investigate the molecular and electronic characteristics of EB, FEN, and their proposed association complex. Molecular structures and electronic properties were visualized using GaussView 6.0. The ground-state geometries of EB and FEN were optimized using the APFD density functional with the LANL2DZ basis set. EB was considered with a net charge of −1 and singlet multiplicity, whereas FEN was considered with a net charge of +1 and singlet multiplicity. The proposed EB–FEN complex was optimized at the same computational level using the LANL2DZ basis set, with a net charge of 0 and singlet multiplicity. Geometry optimizations were performed using tight convergence criteria, an ultrafine integration grid, and enhanced accuracy for two-electron integrals.

[Int = (Ultrafine, Acc2E = 12)]. We then performed frequency calculations on the optimized structures to verify their geometries.

The electronic energies obtained from the optimized structures were used to calculate the interaction energy according to:Δ*E* = *E*_complex_ − (*E*_EB_ + *E*_FEN_)where *E*_complex_, *E*_EB_, and *E*_FEN_ represent the calculated electronic energies of the optimized EB–FEN complex, EB, and FEN, respectively. We used the interaction energy to evaluate the energetic favorability of the proposed molecular association.

We obtained the dipole moments and isotropic polarizabilities of EB, FEN, and the EB–FEN complex from the optimized structures and compared them to assess changes in electronic properties upon complex formation.

Frontier molecular orbital analysis was performed by examining the highest occupied molecular orbital (HOMO) and lowest unoccupied molecular orbital (LUMO) of EB, FEN, and the optimized EB–FEN complex. The corresponding frontier orbital energies and HOMO–LUMO energy gaps were determined, and the orbital distributions were visualized using GaussView 6.0.

We generated molecular electrostatic potential (MEP) maps for the optimized structures to characterize the electrostatic potential distribution over the molecular surfaces. The MEP surfaces were used to identify electron-rich and electron-deficient regions and to examine their spatial complementarity between EB and FEN.

The optimized structures, HOMO and LUMO distributions, MEP maps, and selected calculated electronic parameters were used collectively to provide a molecular-level interpretation of the experimentally observed EB–FEN interaction.

### 2.5 Method validation

The proposed spectrofluorimetric quenching method was validated in accordance with the ICH requirements. The validation criteria included linearity, detection and quantification limits, and accuracy, precision, selectivity, and robustness.

### 2.6 Applications include commercial tablets, spiked plasma, and spiked milk

#### 2.6.1. Pharmaceutical formulation analysis

FEN quantification in pharmaceutical tablet formulations was performed using the established EB fluorescence-quenching approach. After weighing and finely powdering ten pills, 150 mg of FEN was added to a 100 mL volumetric flask. The powder was sonicated in 10 ml of ethanol for 20 minutes to achieve complete dissolution. The solution was subsequently filtered through a 0.45 µm membrane filter, diluted to the required concentration with distilled water, and further diluted to the desired working concentration. Following the procedures outlined in the linearity and calibration graphs, the five recommended samples were further diluted in distilled water and subjected to quantitative analysis.

#### 2.6.2 Analysis of spiked bovine plasma samples

FEN in biological matrices was determined using matrix-matched sample preparation and calibration. Aliquots of 1.0 mL of drug-free plasma were supplemented with specific quantities of standard FEN solution to achieve concentrations of 0.050, 0.100, 0.150, and 0.200 µg mL^−1^. Protein precipitation was conducted by introducing acetonitrile (plasma : acetonitrile, 1 : 2, v/v), followed by vortex mixing and centrifugation at 5000 rpm for 15 minutes at 4 °C. The transparent supernatants were isolated and evaporated to dryness using a moderate nitrogen stream. The residue was reconstituted in 2.0 mL of a 50 : 50 (v/v) ethanol–water solution, vortex-mixed, and filtered through 0.22 µm syringe filters. The filtrates were quantitatively transferred into 10.0 mL volumetric flasks and analyzed using the standard procedure with erythrosine B.^47–49^

A matrix-matched calibration curve was established over 0.050–0.200 µg mL^−1^ by analyzing spiked plasma samples under identical conditions. Blank plasma samples were analyzed concurrently to adjust for background and reduce potential matrix interference.

#### 2.6.3 Analysis of spiked bovine milk

Milk proteins are divided into two major classes. Casein (thermally stable) represents approximately 80% of the total milk proteins, and whey proteins (thermally labile) represent the remaining 20%. Casein exists in colloidal form at the natural pH of milk (6.6) and precipitates on acidification. Maximum precipitation occurs at its isoelectric point (pH 4.6), where the net charge is zero. The procedure for preparing milk samples was slightly modified. Protein precipitation was done by adding 0.6 mL of glacial acetic acid to the milk sample. 0.9 mL of 0.1 N EDTA was then added. EDTA chelates calcium ions and disrupts casein micelles, improving matrix cleanliness. The mixture was centrifuged at 5000 rpm for 15 min to separate the precipitated proteins.^50^

The clear supernatant was carefully removed and evaporated to dryness at 60 °C. The residue was dissolved in ethanol–water (50 : 50, v/v), vortexed until completely dissolved, and centrifuged to remove any residual particles or lipid droplets. For spectrofluorimetric analysis, a 1.0 mL aliquot of the prepared extract was quantitatively transferred into a 10.0 mL volumetric flask and treated with erythrosine B as described in the general procedure. A matrix-matched calibration curve was constructed by spiking drug-free milk samples with FEN at concentrations ranging from 0.05 to 2.0 µg mL^−1^, using the same procedure. Blank milk samples were processed simultaneously and used for background correction to remove potential matrix interference.

## 3 Results and discussion

### 3.1 Spectral characteristics and studies on complex formation

EB is a xanthene-based fluorophore whose photophysical behavior is strongly influenced by the presence of four iodine atoms incorporated into its conjugated aromatic skeleton.^31^ These heavy atoms enhance spin–orbit coupling and promote intersystem crossing while maintaining an intense fluorescence signal in the visible region. Consequently, EB has been widely used as a fluorescent probe in analytical chemistry because of its high molar absorptivity, intense emission, and pronounced susceptibility to fluorescence quenching upon association with suitable analytes.^51^ In the present study, EB was selected as the fluorescent probe because its excitation and emission wavelengths (527 and 544 nm, respectively) lie within the visible region, where interference from endogenous biological constituents is considerably lower than in the ultraviolet region. In addition, the ionizable carboxylic and phenolic groups of EB enable pH-dependent interactions with protonated analytes, making it suitable for investigating its association with FEN under acidic conditions.

The fluorescence spectrum of EB recorded under the optimized experimental conditions exhibited a well-defined emission maximum at 544 nm upon excitation at 527 nm (Fig. 1A). The fluorescence signal remained stable throughout the measurement period, indicating that the selected experimental conditions preserved the intrinsic fluorescence characteristics of EB.

**Fig. 1:**
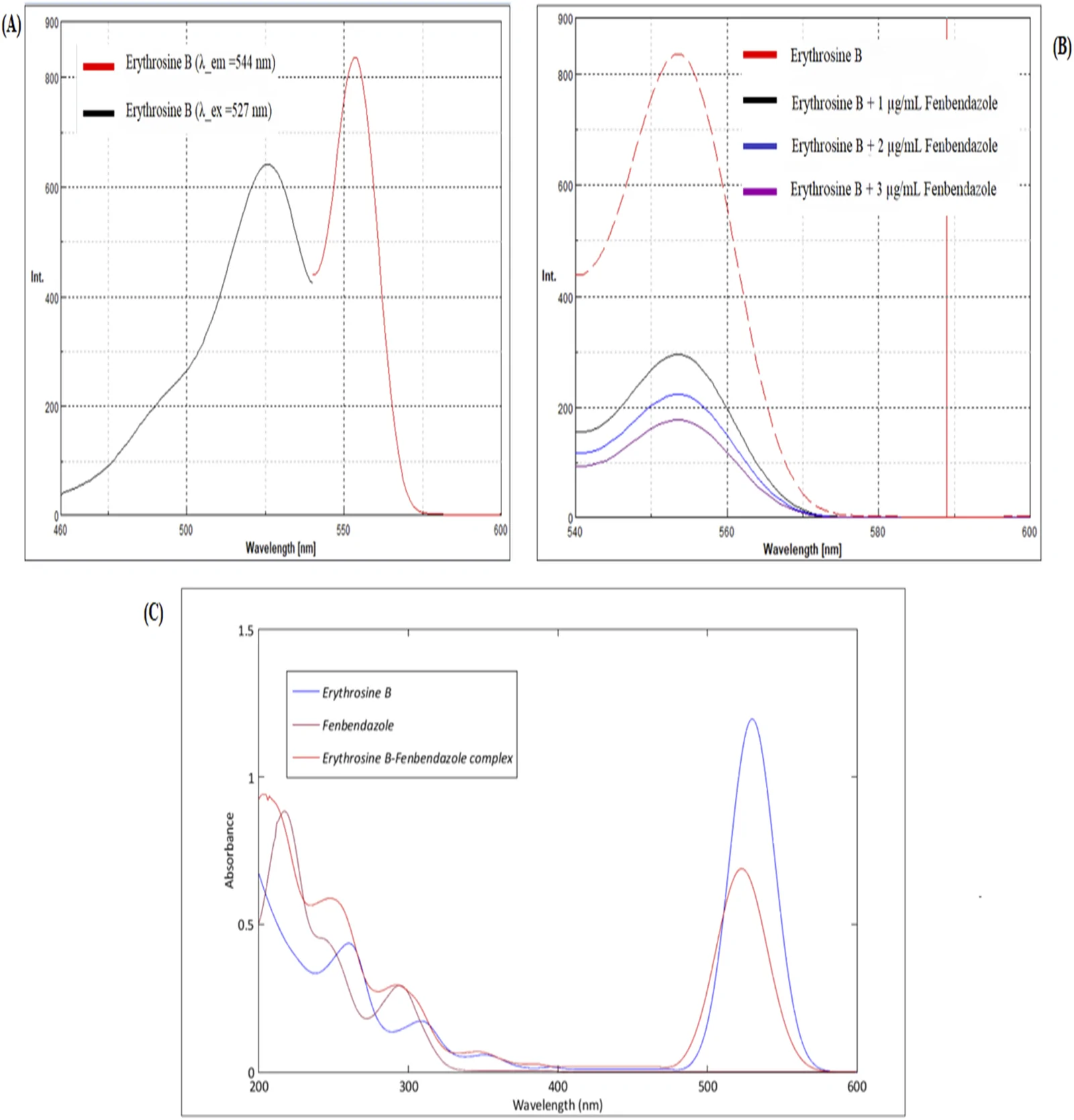
Spectroscopic characterization of the EB–FEN system. (A) EB emission spectrum at 527 nm excitation. (B) EB fluorescence spectra at increasing FEN concentrations. (C) UV-visible absorption spectra of EB, FEN, and their mixture.

Upon gradual addition of FEN (0.05–3.0 µg mL^−1^), a progressive decrease in the fluorescence intensity of EB was observed without any appreciable shift in the emission maximum or distortion of the spectral profile (Fig. 1B). The preservation of the emission wavelength together with the progressive decrease in fluorescence intensity indicates that FEN alters the fluorescence response of EB without causing a major change in its emission profile. The nature of this quenching process was subsequently investigated using temperature-dependent Stern–Volmer analysis.

To further characterize the interaction between EB and FEN, the UV-visible absorption spectra of EB, FEN, and their mixture were compared (Fig. 1C). The experimental spectrum of the EB–FEN mixture exhibited clear hypochromic changes together with slight spectral perturbations relative to the individual components, indicating a change in the electronic environment upon molecular association.

Furthermore, to determine whether the observed spectral changes could be explained solely by the additive contribution of the individual components, the experimental absorption spectrum of the EB–FEN mixture was compared with the mathematically summed spectrum obtained from the individual EB and FEN spectra recorded under identical experimental conditions (Fig. S1). A pronounced deviation was observed between the experimental mixture spectrum and the mathematically summed spectrum. This non-additive spectral behavior indicates that the observed response cannot be explained solely by the arithmetic summation of the individual components and provides additional evidence of an interaction between EB and FEN.

The potential contribution of the inner filter effect (IFE) to the observed fluorescence decrease was also carefully evaluated by comparing the UV-visible absorption spectrum of FEN with the excitation and emission wavelengths of EB (527 and 544 nm, respectively) (Fig. S2). The UV-visible spectrum of FEN showed no appreciable absorbance at either 527 or 544 nm throughout the investigated concentration range. Thus, FEN does not significantly attenuate the excitation radiation at 527 nm or reabsorb the emitted fluorescence at 544 nm. Consequently, both primary and secondary IFE contributions from FEN are considered negligible under the experimental conditions. Therefore, the progressive decrease in EB fluorescence upon addition of FEN cannot reasonably be attributed to an optical absorption artifact.

Collectively, the fluorescence and UV-visible spectral findings, together with the non-additive behavior of the mixture spectrum and the negligible contribution of IFE, demonstrate that the observed fluorescence response is associated with the interaction of FEN with EB rather than with an optical absorption artifact. The nature and mechanism of this interaction were subsequently investigated through temperature-dependent Stern–Volmer analysis, thermodynamic and binding studies, Job's method of continuous variation, and DFT calculations.

### 3.2 Mechanistic investigations

Following the spectral evidence, mechanistic studies were performed to elucidate the nature of the interaction between EB and FEN. We systematically investigated the quenching process by evaluating the IFE, temperature-dependent Stern–Volmer analysis, binding and thermodynamic studies, and Job's method of continuous variation. These complementary experiments provided evidence for the quenching mechanism, binding affinity, spontaneity of complex formation, and interaction stoichiometry, further supporting the proposed ground-state association between EB and FEN.

#### 3.2.1 Quenching mechanism and Stern–Volmer analysis

Temperature-dependent Stern–Volmer studies were performed at 298, 303, and 313 K to investigate the fluorescence quenching behavior of EB by FEN (Fig. 2A). The fluorescence data exhibited excellent linearity throughout the studied concentration range (*R*^2^ > 0.999) according to the classical Stern–Volmer equation:*F*_0_/*F* = 1 + *K*_sv_[*Q*]where *F*_0_ and *F* are the fluorescence intensities of EB in the absence and presence of FEN, respectively; *K*_sv_ is the Stern–Volmer quenching constant; and [*Q*] represents the molar concentration of FEN.

**Fig. 2:**
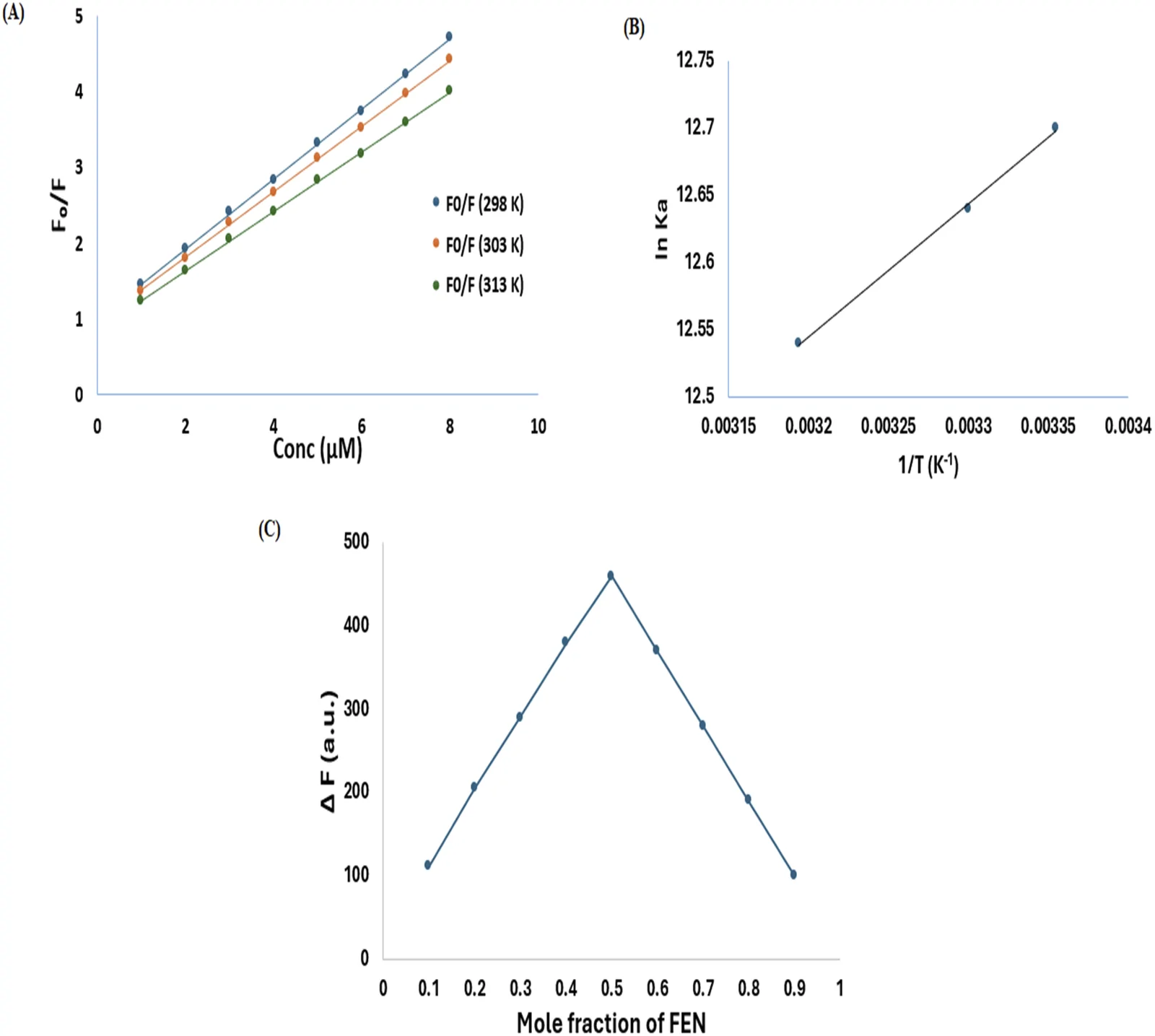
Mechanistic investigation of the EB–FEN interaction. (A) Stern–Volmer plots at 298, 303, and 313 K. (B) Van't Hoff plot of ln *K*_a_*versus* 1/*T*. (C) Job's continuous variation plot of the EB–FEN system.

The calculated Stern–Volmer constants gradually decreased from 3.54 × 10^5^ M^−1^ at 298 K to 3.25 × 10^5^ M^−1^ at 303 K and 3.09 × 10^5^ M^−1^ at 313 K (Table 1). The progressive decrease in *K*_SV_ with increasing temperature indicates reduced stability of the fluorophore-analyte association upon heating, which is characteristic of predominantly static quenching. In contrast, dynamic quenching is generally favored at elevated temperatures because of enhanced molecular diffusion.

**Table 1:** Shows the thermodynamic parameters (Δ*H*, Δ*S*, and Δ*G*) and temperature-dependent association constants (*K*_a_) for the erythrosine B–FEN binding interaction

Temp. (K)	*K*_sv_ × (10^5^ M^−1^)	*K*_a_ × (10^5^ M^−1^)	Δ*G* (kJ mol^−1^)	Δ*H* (kJ mol^−1^)	Δ*S* (J mol^−1^ K^−1^)
298	3.54	3.28	−31.47	−8.21	78.04
303	3.25	3.09	−31.84		
313	3.06	2.79	−32.63		

Additional evidence was obtained from the calculated bimolecular quenching rate constants (*K*_Q_), which exceeded 10^15^ M^−1^ s^−1^, greatly surpassing the diffusion-controlled limit in aqueous media (approximately 10^10^ M^−1^ s^−1^). Such values are inconsistent with diffusion-controlled collisional quenching alone and provide further support for a predominantly static quenching mechanism involving formation of a non-fluorescent EB–FEN ground-state association.

#### 3.2.2 Thermodynamic and binding investigation

The binding interaction between EB and FEN was further evaluated using the association constants (*K*_a_) determined at the investigated temperatures through van't Hoff analysis (Fig. 2B). The association constants (*K*_a_) were 3.28 × 10^5^, 3.09 × 10^5^, and 2.79 × 10^5^ M^−1^ at 298, 303, and 313 K, respectively (Table 1). The gradual decrease in *K*_a_ with increasing temperature indicates reduced stability of the associated species upon heating, consistent with the temperature-dependent Stern–Volmer behavior.

The linear van't Hoff relationship between ln *K*_a_ and 1/*T* was used to determine the thermodynamic parameters of the association. The negative enthalpy change (Δ*H* = −8.21 kJ mol^−1^) indicates that the association process is exothermic. The Gibbs free energy changes obtained over the investigated temperature range were negative, indicating that the association is thermodynamically spontaneous under the experimental conditions. A positive entropy change (Δ*S* = +78.04 J mol^−1^ K^−1^) was also obtained, indicating a favorable entropic contribution to the association process. This behavior may be associated, at least in part, with the release of structured solvent molecules upon association.

The logarithmic binding relationship was additionally examined to estimate the apparent number of binding sites:Log[(*F*_0_ − *F*)/*F*] = log *K*_a_ + *n* log[*Q*]

The logarithmic binding plot at 298 K exhibited excellent linearity, with an *R*^2^ value of 0.9995 and a slope (*n*) of 0.992. The value of *n* being very close to unity indicates approximately one predominant binding site under the investigated conditions. The stoichiometric relationship between EB and FEN was subsequently examined independently using Job's method of continuous variation.

#### 3.2.3 Complex formation and stoichiometry

The stoichiometry of the EB–FEN association was investigated using Job's method of continuous variation (Fig. 2C). A well-defined maximum was observed at a FEN mole fraction of 0.5, corresponding to a 1 : 1 molar ratio between EB and FEN. Thus, one EB molecule interacts predominantly with one FEN molecule under the optimized experimental conditions.

The Job's plot result closely agrees with the apparent binding-site number obtained from the logarithmic binding analysis (*n* = 0.992), providing complementary evidence for the predominance of a 1 : 1 association. The molecular features underlying this association, including the possible electrostatic and hydrogen-bonding contributions, were further investigated using DFT calculations.

### 3.3 Density functional theory (DFT) investigation of the interaction mechanism

To obtain molecular-level insight into the EB–FEN interaction, density functional theory (DFT) calculations were performed. The geometries of the individual species and their corresponding complex were optimized, and the optimized structures, molecular electrostatic potential (MEP) surfaces, frontier molecular orbitals (HOMO and LUMO), and proposed interaction model are presented in Fig. 3–5. The optimized EB–FEN complex exhibited a lower calculated electronic energy than the corresponding isolated components. The interaction energy was calculated according to: Δ*E* = *E*_Complex_ − (*E*_EB_ + *E*_FEN_).where *E*_Complex_, *E*_EB_, and *E*_FEN_ represent the calculated electronic energies of the optimized EB–FEN complex, EB, and FEN, respectively. The calculated raw interaction energy was −16.92 kcal mol^−1^. In comparison, the BSSE-corrected interaction energy was −13.49 kcal mol^−1^, indicating that the association remains energetically favorable after correction for basis-set superposition error. The absence of imaginary frequencies for the optimized structures further confirmed that the calculated geometries correspond to stable minima on the potential-energy surface. The molecular electrostatic potential (MEP) surfaces of EB and FEN provided insight into the electrostatic complementarity of the interacting species (Fig. 4). EB exhibited pronounced negative electrostatic potential regions, predominantly associated with its oxygen-containing functionalities. In contrast, FEN displayed comparatively positive electrostatic potential around its protonatable region. This complementary electrostatic potential distribution provides a plausible structural basis for electrostatic attraction between the two species under the acidic experimental conditions.

**Fig. 3:**
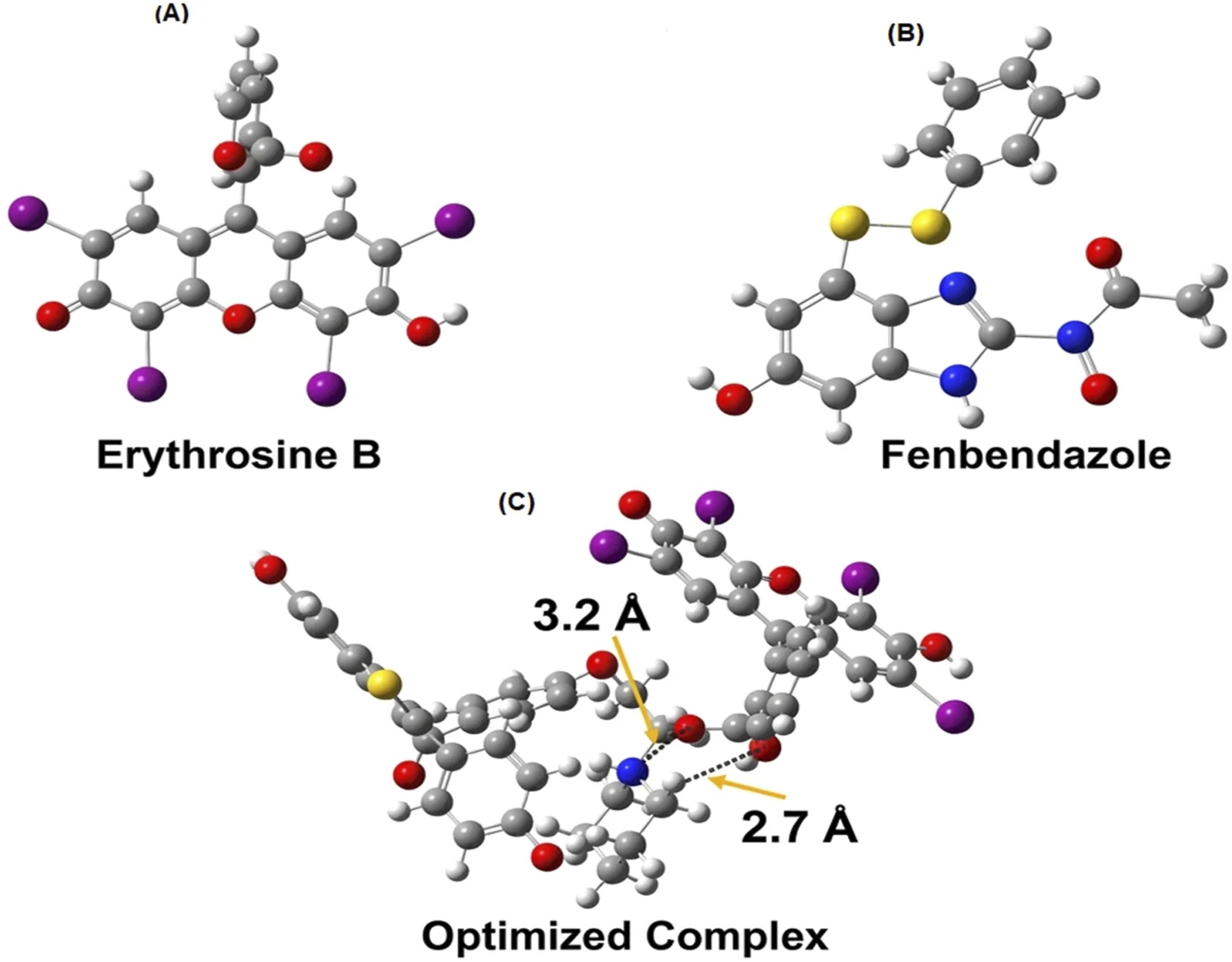
DFT-optimized molecular structures of the EB–FEN system. (A) Erythrosine B. (B) FEN. (C) EB–FEN complex.

**Fig. 4:**
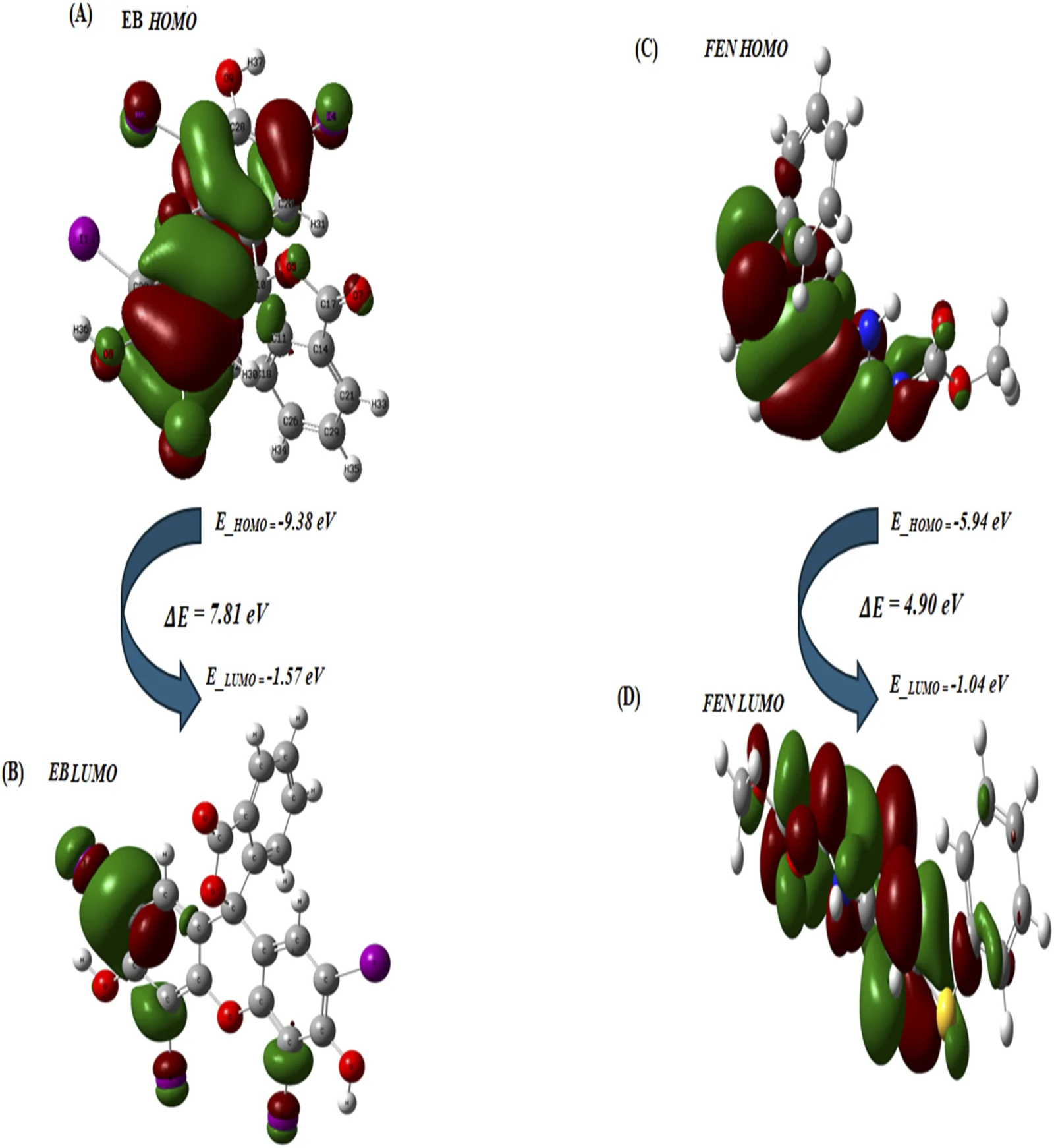
Frontier molecular orbital distributions of (A) HOMO and (B) LUMO of EB, and (C) HOMO and (D) LUMO of FEN.

**Fig. 5:**
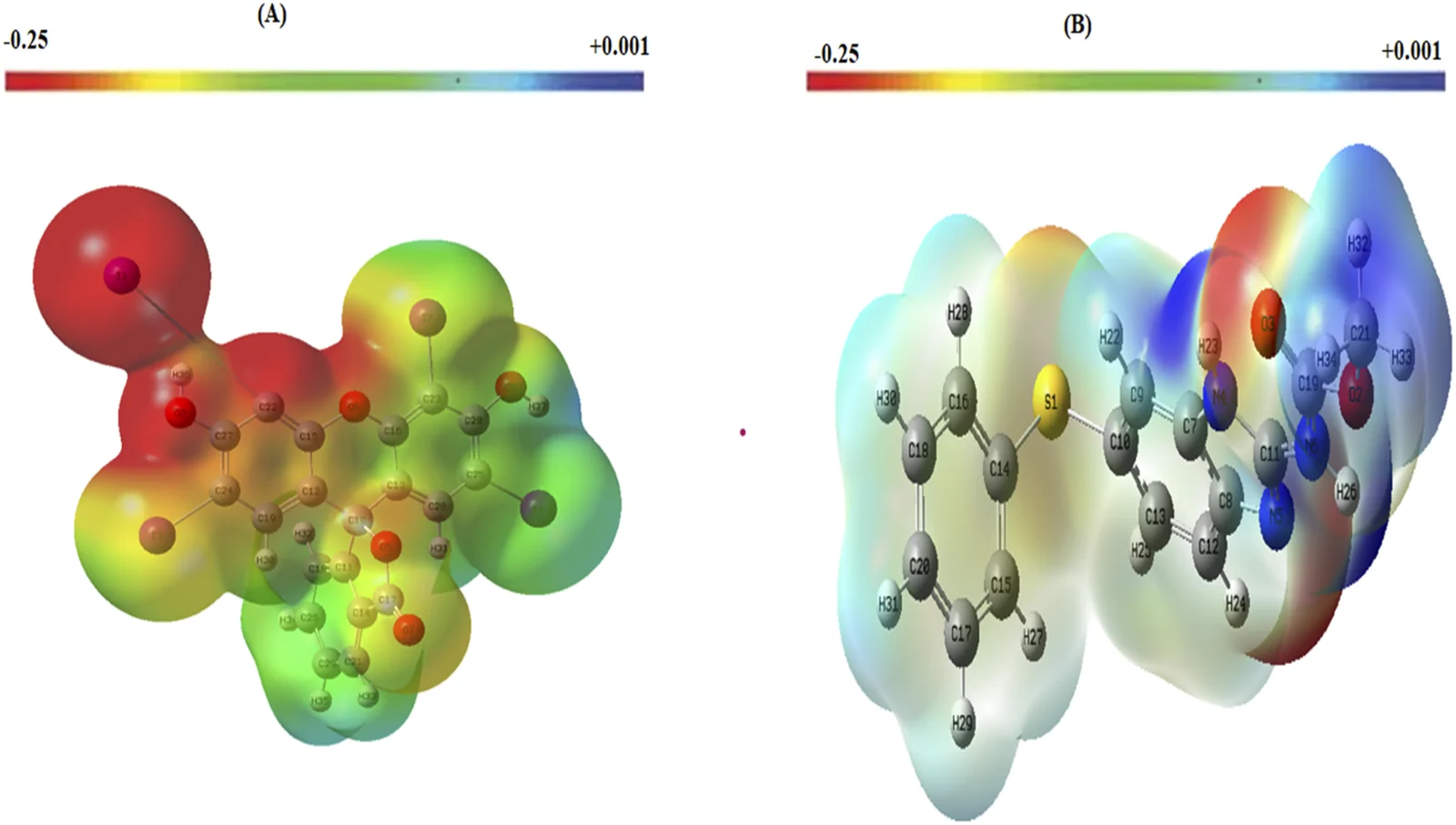
MEP Maps of EB (A) and FEN (B) showing complementary electrostatic regions.

The frontier molecular orbitals of EB, FEN, and the optimized EB–FEN complex were also examined (Fig. 5). The isolated molecules showed distinct HOMO and LUMO distributions, reflecting their different electronic characteristics. For EB, the frontier orbitals were predominantly associated with its extended conjugated xanthene framework, whereas the frontier orbital contributions of FEN involved its benzimidazole and carbamate-containing regions. Upon complex formation, orbital localization and electron-density distributions changed, indicating electronic redistribution accompanying molecular association. The calculated HOMO–LUMO gaps of the isolated EB and FEN were approximately 7.81 and 4.91 eV, respectively. The optimized complex exhibited a substantially smaller HOMO–LUMO gap of 2.71 eV, indicating a pronounced change in the electronic structure upon association. This reduction is consistent with increased electronic delocalization and redistribution within the associated system. Nevertheless, the HOMO–LUMO gap alone should not be regarded as direct evidence of intermolecular charge transfer; rather, the changes in frontier-orbital localization and electron-density distribution provide complementary evidence of electronic interaction between EB and FEN.

The optimized EB–FEN geometry revealed close contacts between complementary functional groups. In particular, the NH moiety of FEN was oriented toward an oxygen atom of the carboxylate group of EB, supporting the possibility of hydrogen-bond formation in addition to electrostatic interactions. These non-covalent contacts provide a plausible structural basis for stabilization of the associated species and are consistent with the favorable calculated interaction energy. The calculated dipole moments and polarizabilities also changed upon complex formation (Table 2), indicating electron-density redistribution following EB–FEN association. The relatively high polarizability of EB can be attributed to its extended π-conjugated xanthene framework and iodine substituents, which contribute to its electronic responsiveness.

**Table 2:** DFT-calculated electronic properties of EB, FEN, and the optimized EB–FEN ion-pair complex, together with the calculated raw and BSSE-corrected complexation energies

Parameter	EB	FEN	Complex	Δ*E* (complexation)
Charge	−1	1	0	—
Spin	Singlet	Singlet	Singlet	—
Method/basis	APFD/LANL2DZ	APFD/LANL2DZ	APFD/LANL2DZ	—
*E* (Hartree)	−1187.623890	−1291.79124858	−2479.442100	−0.02696142
Δ*E*_raw_ (kcal mol^−1^)				−16.92
BSSE (kcal mol^−1^)				3.43
Δ*E*_CP_ (kcal mol^−1^)				−13.49
HOMO–LUMO gap (eV)	7.81	4.9171	2.7143	—
Dipole moment (D)	35.9176	5.1883	26.924	—
Polarizability (a.u.)	408.117	235.34	663.21	—
Imaginary freq.	0	0	0	—

Overall, the DFT results provide a molecular-level rationale for the experimentally observed EB–FEN association. The favorable BSSE-corrected interaction energy, complementary MEP distributions, hydrogen-bonding geometry, and changes in frontier-orbital distributions collectively indicate that electrostatic attraction and hydrogen bonding are plausible contributors to complex stabilization. These computational findings complement the spectroscopic, thermodynamic, and stoichiometric results and provide a coherent molecular interpretation of the observed fluorescence quenching.

### 3.4 Optimization of experimental conditions

The experimental variables affecting fluorescence quenching were systematically optimized to maximize sensitivity, reproducibility, and complex stability. Parameters influencing ion–pair formation, including the buffer system, solution pH, buffer volume, EB concentration, diluting solvent, and reaction time, were investigated individually while keeping all other experimental variables constant.

#### 3.4.1 Effect of buffer system and pH

The choice of buffer plays an important role in controlling both the ionization state of the interacting species and the stability of the fluorescence signal. We preliminarily evaluated several commonly used acidic buffer systems. Britton–Robinson buffer produced the highest and most reproducible quenching response over the investigated pH range and was therefore selected for further experiments.

We then investigated the effect of pH between 2.5 and 6.0 using B–R buffer (Fig. 6A). The quenching response gradually increased as pH decreased, reaching a maximum at pH 3.0. Under these conditions, FEN exists predominantly in its protonated form, whereas EB remains mainly in its anionic form, favoring electrostatic attraction and formation of a stable ion-pair complex. At higher pH values, partial deprotonation of FEN reduced electrostatic interactions, thereby decreasing quenching efficiency. Therefore, 2.0 mL of Britton–Robinson buffer at pH 3.0 was selected as the optimum buffering system for all subsequent experiments.

**Fig. 6:**
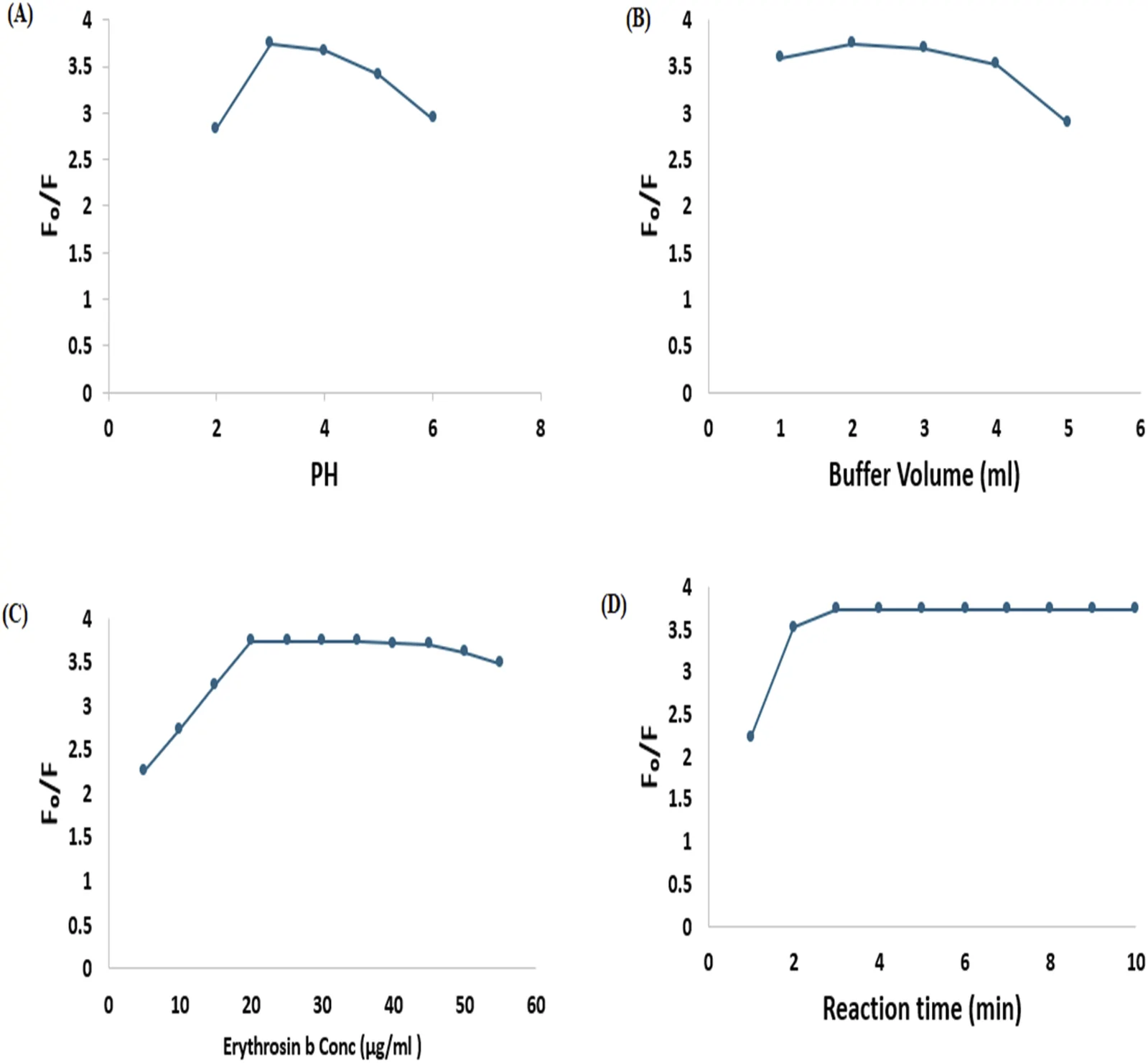
Optimization of the experimental conditions for the EB–FEN fluorescence quenching system. (A) Effect of pH. (B) Effect of buffer volume. (C) Effect of EB concentration. (D) Effect of reaction time.

#### 3.4.2 Effect of buffer volume

The influence of buffer volume was investigated over 0.5–3.0 mL (Fig. 6B). Increasing the buffer volume enhanced fluorescence quenching up to 2.0 mL, beyond which no significant improvement was observed. Larger buffer volumes slightly decreased analytical response, most likely due to dilution of the reacting species. Accordingly, 2.0 mL was selected as the optimum buffer volume.

#### 3.4.3 Effect of EB concentration

The EB concentration was optimized to provide adequate fluorescence intensity while maintaining maximum analytical sensitivity. Increasing the dye concentration progressively enhanced the initial fluorescence signal. Consequently, it increased the quenching response until a final concentration of 20.0 µg mL was reached (Fig. 6C). Above this concentration, quenching efficiency did not increase appreciably, indicating saturation of the available binding sites. Therefore, 20.0 µg mL was selected for subsequent measurements.

#### 3.4.4 Effect of reaction time

The time required for complex formation was evaluated by monitoring fluorescence intensity immediately after mixing the reagents. The quenching process reached completion within 3 min at room temperature. It remained essentially unchanged for at least 30 min (Fig. 6D), indicating rapid formation and satisfactory stability of the EB–FEN complex during the analytical measurement. Consequently, we adopted a reaction time of 3 min throughout the study.

Under the optimized conditions—2.0 mL Britton–Robinson buffer (pH 3.0), 20.0 µg mL EB, ethanol–water (1 : 10, v/v) as diluent, and a reaction time of 3 min—the proposed system provided rapid, reproducible, and efficient formation of the EB–FEN ion-pair complex, resulting in highly sensitive fluorescence quenching suitable for subsequent quantitative analysis.

### 3.5 Analytical method validation

The optimized experimental parameters were validated in accordance with ICH Q2(R2) guidelines, and the suitability of the proposed analytical procedure was confirmed for pharmaceutical analysis and bioanalytical applications.

A linear relationship was established between the fluorescence quenching response and FEN concentration, expressed by the regression equation:

*F*_0_/*F* = 0.354[C] + 1.0251, where [C] represents the drug concentration in µg mL. The method showed good linearity over 0.05–3.0 µg mL^−1^, with a correlation coefficient (*r*^2^) of 0.9996, confirming a proportional response within the studied range. This working range adequately covers the expected FEN levels encountered in dosage forms and biological systems after regular administration. Sensitivity evaluation showed a limit of detection (LOD) of 0.014 µg mL and a limit of quantification (LOQ) of 0.042 µg mL, demonstrating the method's high sensitivity for detecting and quantifying trace analyte levels. These sensitivities are comparable to, and in many cases better than, those reported for conventional chromatographic techniques, with the added benefits of operational simplicity and lower cost. We conducted repeatability and intermediate precision studies to evaluate the method's precision. Intra-day precision was determined at three concentration levels with nine replicate measurements, and the relative standard deviation (RSD) was found to be 0.570%, indicating excellent repeatability within the acceptable limit of ≤2.0%. The inter-day precision was determined over three consecutive days, yielding an RSD of 0.748%, which further confirms the robustness and reliability of the analytical procedure under routine laboratory conditions.

The obtained recovery results confirmed the accuracy of the proposed method, with a mean recovery of 99.19 ± 0.565%. The recoveries were within the range of 98–102%, demonstrating close agreement between the measured and nominal concentrations and indicating no appreciable systematic error. These results confirm the method's satisfactory trueness. Furthermore, the robustness of the method was evaluated by deliberately introducing small variations in critical experimental parameters, including buffer pH (±0.1 units), reagent concentration (±5%), and reaction time (±0.5 min). Such minor variations produced no significant changes in the analytical response, with consistent recovery values and % RSD values below 2%. These findings show that the method is sufficiently robust to maintain reliable analytical performance under small, unavoidable variations in experimental conditions (Table 3).

**Table 3:** Regression and validation data for the proposed quenching method's FEN calculation

Parameters	FEN
Excitation wavelength (nm)	527 nm
Emission wavelength (nm)	544 nm
Linearity range (µg ml^−1^)	0.05–3 µg ml
Determination coefficient (*r*^2^)	0.9997
Intercept	1.025
Slope	0.354
LOD (µg mL^−1^)	0.014 µg ml
LOQ (µg mL^−1^)	0.043 µg ml
Accuracy^a^ (% *R*)	99.192
Precision^b^ (% RSD)	
Repeatability	0.570
Intermediate precision	0.748
Robustness (% RSD)	
Buffer (pH)	0.892
Reagent conc.(µg ml^−1^)	0.986
Reaction time (min)	1.143

a Average of nine determinations (three concentrations repeated three times).

b RSD of nine determinations (three concentrations repeated three times).

Selectivity was evaluated by assessing potential interference from common substances encountered in pharmaceutical formulations and biological matrices (Fig. 7). Potential interferents, including inorganic ions, amino acids, and plasma constituents, were evaluated over a wide range of FEN concentrations (up to 10-fold). The quenching effect observed for these species was no more than 3%, indicating minimal interference and high selectivity.^52–54^ In conclusion, the validation results indicate that the proposed method is reliable, sensitive, and suitable for routine analysis. However, additional studies on forced degradation under ICH-recommended stress conditions would help develop a stability-indicating version of the method. Such studies provide a better understanding of the correlations between degradation behavior and key validation parameters, including specificity, selectivity, and sensitivity, thereby enhancing the applicability of these parameters in pharmaceutical quality control.

**Fig. 7:**
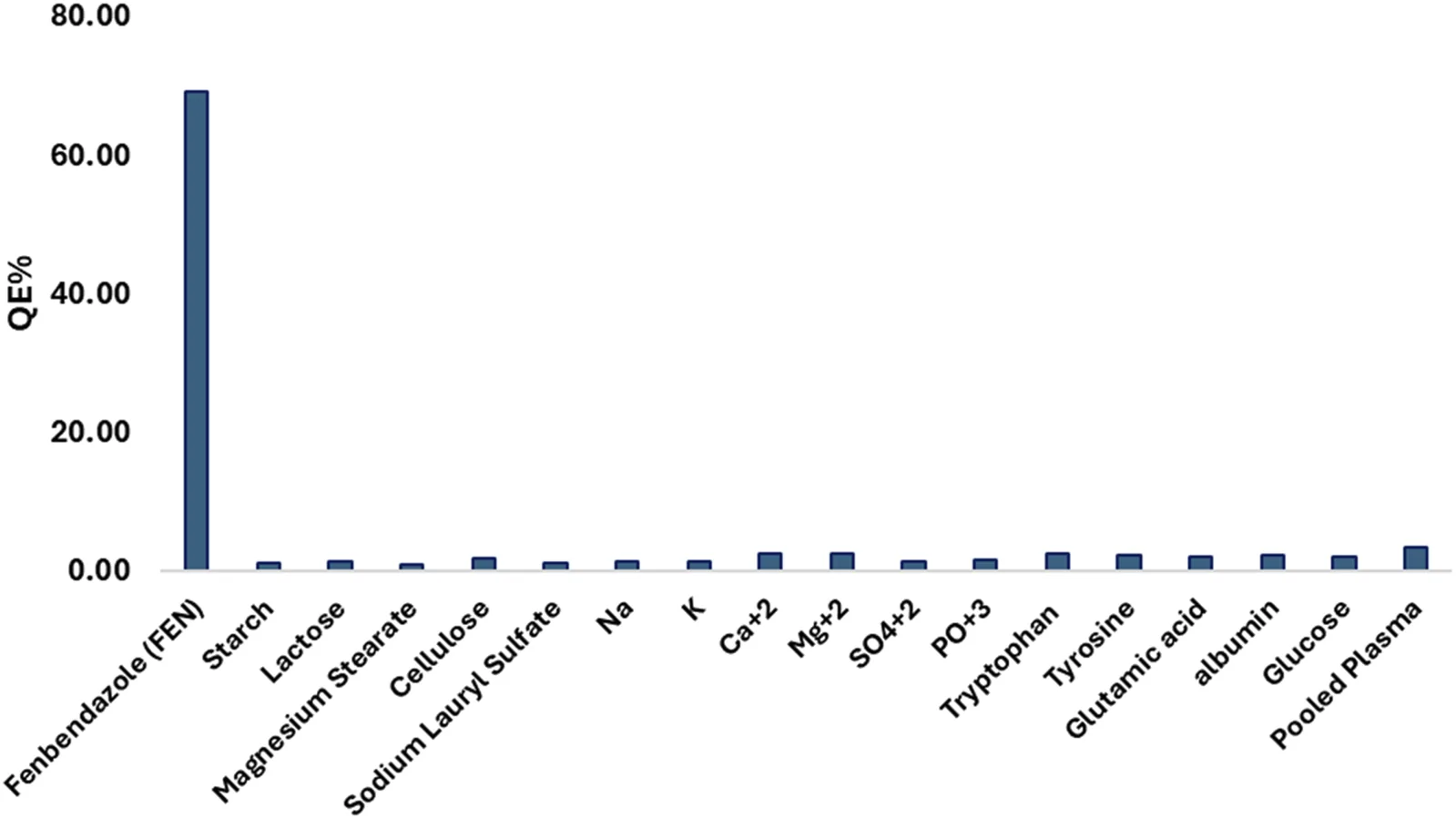
Assessing the selectivity of the newly designed fluorescence quenching method. The graphic compares the quenching efficiency (%) of FEN (2 µg ml^−1^) with those of other potentially interfering chemicals at tenfold excess concentrations, including co-administered drugs, pharmaceutical excipients, amino acids, inorganic salts, and blank plasma samples.

### 3.6 Application to pharmaceutical formulation, spiked bovine plasma and bovine milk

The validated spectrofluorimetric approach was successfully applied to pharmaceutical dosage forms and veterinary matrices, including spiked bovine plasma and milk. The emphasis was on confirming the approach's selectivity and removing interference using blank samples and matrix-matched calibration.

#### 3.6.1 Analysis of pharmaceutical formulation

The method was successfully applied to determine FEN in its commercial dosage form. The assay results agree closely with the labeled claim, confirming the procedure's accuracy. To assess selectivity, placebo solutions containing all excipients except the active ingredient were evaluated in the same manner. The negligible matched-blank results indicated no interference from common excipients. To verify the method's accuracy, the standard addition procedure was used. The recovered values were within acceptable ranges, and the relative standard deviations were modest, suggesting that excipient interference was effectively removed and that the procedure was applicable for routine quality control analysis (Table 4).

**Table 4:** Evaluation of FEN in pharmaceutical tablets using the suggested quenching technique and statistical comparison with the reported method

Parameters	Proposed method	Reported method
Number of measurements	5	5
Mean% recovery^a^	99.283	98.561
% RSD	1.182	1.561
Variance	1.376	2.369
Students' *T*-test^b^	0.834 (2.306)	—
*F*-Value^b^	1.722 (6.388)	—

a Average of five determinations.

b The values in parentheses are tabulated values of *t* and *F* at(*P* = 0.05).

#### 3.6.2 Analysis of spiked bovine plasma

The applicability of the approach to biological matrices was investigated with spiked bovine plasma samples. Samples were evaluated after protein precipitation and appropriate sample preparation utilizing a matrix-matched calibration method. Blank bovine plasma samples (drug-free) were prepared and run in parallel to assess potential interference. Under these conditions, the blank plasma showed little fluorescence, indicating that the endogenous plasma component did not interfere with the measurement. The spiked samples demonstrated strong, consistent recoveries across the concentration range studied, with low variability, indicating efficient extraction and accurate measurement. These results provide evidence that matrix effects were successfully controlled (Table 5).

**Table 5:** Application of the proposed quenching method for spiked bovine plasma and milk samples

Samples	FEN added amount (ng ml^−1^)	FEN found amount (ng ml^−1^)	FEN^a^ (% *R*)
Plasma	50	47.9	95.800
	500	489	97.800
	1500	1484	98.933
	2000	1971	98.550
	Mean ± % RSD		97.77 ± 1.43
Milk	50	48.1	96.200
	500	489	97.800
	1500	1488	99.200
	2000	2009	100.450
	Mean ± % RSD		98.41 ± 1.86

a Average of three determinations.

#### 3.6.3 Analysis of bovine milk

We also tested the procedure on bovine milk samples to evaluate performance. We evaluated selectivity by comparing blank milk samples with spiked samples. The blank matrix showed no significant signal, suggesting that proteins, lipids, and other milk constituents did not interfere with the fluorescence measurements.

The spiked milk samples showed good recoveries and acceptable precision, demonstrating the sample preparation method's efficiency in eliminating matrix interference and enabling precise determination. The results suggest the approach's applicability to determining FEN residues in food matrices, validating selectivity and improving overall performance. Blank matrices (placebo, drug-free bovine plasma, and blank milk) provided strong evidence that excipients and endogenous components did not interfere with the test. All blank studies yielded negligible signals, thereby demonstrating the approach's great selectivity. Overall, the technique showed good accuracy, precision, and robustness across all matrices studied. The consistent results and effective exclusion of matrix interferences through blank-curve analysis demonstrate the method's applicability for routine FEN determination in pharmaceutical formulations, veterinary biological samples, and food matrices, without complex separation techniques (Table 5).

### 3.7 Evaluation of environmental impact and sustainability

The environmental sustainability of the new EB–FEN fluorescence-quenching technology was thoroughly assessed using a multimeric approach based on complementary green, blue, and white analytical chemical methods. The assessment using Green AGREE (AGREEprep), ComplexMoGAPI, CaFRI, BAGI (blue applicability), and RGB12 (white analytical chemistry) provided a thorough evaluation of environmental effects, functional sustainability, and practical applications. The current study strongly supports the conclusion that this integrated strategy is superior to standard chromatographic techniques widely used for FEN detection.^55–67^

#### 3.7.1 AGREEprep

The environmental performance of the sample-preparation procedure was assessed using the AGREEprep metric, which evaluates sample preparation according to the principles of green analytical chemistry. The proposed procedure achieved an AGREEprep score of 0.75, indicating a favorable environmental profile. The corresponding clock-shaped pictogram (Fig. 8a) showed predominantly green segments, reflecting strong compliance with most evaluated sustainability criteria.

**Fig. 8:**
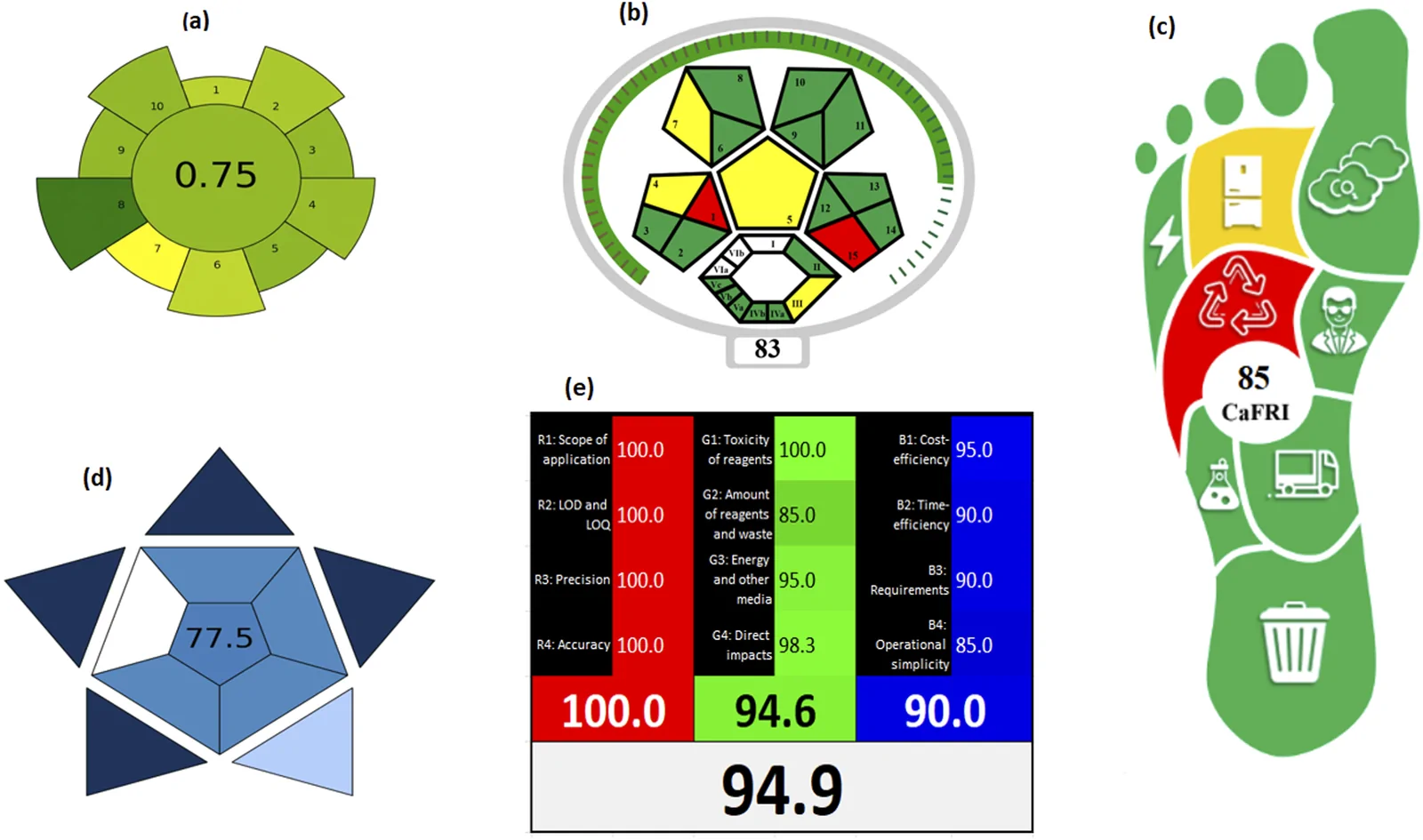
Comprehensive evaluation of the proposed fluorescence quenching method using: (a) analytical Eco-Scale, (b) AGREE metric, (c) CaFRI footprint assessment, (d) BAGI index, and (e) RGB model for White Analytical Chemistry (WAC) assessment.

The favorable score mainly reflected the relatively simple sample-preparation workflow, which involved protein precipitation using a limited amount of acetonitrile, followed by solvent evaporation and reconstitution in an aqueous ethanol solution. Although acetonitrile is less environmentally preferable, its use was limited to the protein-precipitation step and was not required during fluorescence measurement. The subsequent evaporation and reconstitution steps introduce additional solvent handling and energy requirements and therefore represent the main environmental limitations of the sample-preparation procedure.

Nevertheless, the overall procedure avoids liquid–liquid extraction, derivatization, and extensive cleanup steps, thereby reducing the number of handling stages, solvent consumption, and waste generation. The final fluorescence measurement is performed in an aqueous medium, without an organic chromatographic mobile phase. Manual operation and the use of conventional instrumentation resulted in moderate scores for automation, miniaturization, and offline processing. Thus, while the sample-preparation procedure is not completely free from environmentally unfavorable solvents, its limited use of acetonitrile and relatively simple workflow contribute to an overall favorable AGREEprep profile.

#### 3.7.2 ComplexMoGAPI

The ComplexMoGAPI assessment yielded an overall score of 83%, indicating a favorable environmental profile across the different stages of the analytical procedure. The pentagonal pictogram (Fig. 8b) considers sample collection, sample preparation, reagents and solvents, instrumentation, and waste generation.

The proposed method requires relatively small amounts of reagents and solvents, with water representing the major solvent component. The use of acetonitrile during plasma protein precipitation is less favorable; however, it is limited to this pretreatment step. In contrast, the subsequent fluorescence measurement does not require an organic mobile phase, high-pressure pumping, or chromatographic separation. The absence of derivatization and extensive extraction procedures further reduces chemical consumption and waste generation.

The solvent–evaporation step also adds energy and handling requirements that should be considered when interpreting the overall sustainability profile. Nevertheless, the short fluorescence measurement time, room-temperature operation, and avoidance of energy-intensive chromatographic separation contribute positively to the overall ComplexMoGAPI assessment. Therefore, the obtained score reflects a balance between the favorable features of the analytical measurement and the unavoidable environmental burden associated with plasma pretreatment.

#### 3.7.3 CaFRI (Carbon Footprint Reduction Index)

The Carbon Footprint Reduction Index (CaFRI) was used to assess the environmental impact associated with solvent consumption, energy use, and waste generation. The proposed method achieved a high CaFRI score, reflecting a comparatively low carbon footprint relative to conventional chromatographic procedures (Fig. 8c).

The favorable CaFRI performance is primarily related to the fluorescence measurement itself, which does not require an organic mobile phase, high-pressure pumping, column heating, or prolonged chromatographic separation. The short measurement time of approximately 3 min also minimizes instrument operating time and the associated energy consumption. In addition, the relatively small reagent volumes and limited waste generation support a favorable carbon-footprint profile. However, the carbon footprint should not be considered negligible because plasma sample preparation involves acetonitrile for protein precipitation, followed by solvent evaporation and reconstitution. These steps add solvent- and energy-related burdens and therefore limit the proposed procedure from a sustainability perspective. Accordingly, the high CaFRI score reflects the balance between the low-impact fluorescence measurement and the additional resources required for biological-sample pretreatment, rather than the complete elimination of environmental burdens.

#### 3.7.4 BAGI (Blue Applicability Grade Index)

The practical applicability of the proposed method was assessed using the Blue Applicability Grade Index (BAGI). The method achieved a BAGI score of 77.5, exceeding the recommended applicability threshold of 60 and indicating good suitability for routine analytical applications (Fig. 8d).

The method demonstrated favorable analytical performance, including good linearity, sensitivity, precision, and accuracy. Its practical advantages include the use of readily available reagents, relatively simple sample preparation, short reaction and measurement times, and the use of conventional spectrofluorometric instrumentation. The method can therefore provide a relatively high analytical throughput, estimated at more than 10 samples per hour under the optimized workflow.

The main practical limitation concerns biological-sample preparation, particularly the requirement for acetonitrile-mediated protein precipitation and subsequent evaporation and reconstitution. Although these steps increase handling time and resource consumption compared with direct aqueous measurement, they remain simpler than extensive extraction or chromatographic sample-preparation procedures. Overall, the BAGI assessment indicates that the proposed method provides a favorable balance between analytical performance, operational simplicity, and practical applicability.

#### 3.7.5 RGB12 (White Analytical Chemistry) evaluation

We also performed a comprehensive sustainability assessment using the RGB12 model, which integrates analytical performance (Red), environmental impact (Green), and practical applicability (Blue). The proposed method achieved a high overall whiteness score of 94.9% (Fig. 8e), reflecting a favorable balance among these three dimensions. The red component (100%) reflects the method's strong analytical performance, including high linearity, satisfactory precision (% RSD < 2%), and acceptable accuracy with recoveries within the range of 98–102%. The green component (94.6%) reflects the relatively low environmental burden associated with the analytical measurement, including low reagent consumption, an aqueous-based measurement medium, short analysis time, and limited waste generation. Nevertheless, this component is partially affected by the use of acetonitrile during plasma protein precipitation, together with the subsequent solvent–evaporation and reconstitution steps. These operations represent the principal environmental limitations of the sample-preparation procedure and prevent the method from being considered completely solvent-free.

The Blue component (90.0%) reflects the method's favorable practical applicability, including operational simplicity, relatively low cost, short analysis time, conventional instrumentation, and limited sample-preparation requirements. Although plasma pretreatment introduces additional handling steps, the overall workflow remains comparatively simple and does not require chromatographic separation.

Taken together, the AGREEprep, ComplexMoGAPI, CaFRI, BAGI, and RGB12 assessments demonstrate a favorable overall sustainability profile for the proposed EB–FEN spectrofluorometric method. Importantly, the sustainability assessment reflects a balance between the environmental advantages of the fluorescence measurement and the limitations associated with biological-sample pretreatment. The limited use of acetonitrile for protein precipitation, along with solvent evaporation and reconstitution, represents the procedure's main environmental burden. Nevertheless, the absence of an organic chromatographic mobile phase, short analysis time, low reagent consumption during measurement, limited waste generation, and simple instrumentation contribute substantially to the overall favorable sustainability profile. Thus, the proposed method is a comparatively sustainable analytical approach, while further reducing or replacing acetonitrile in sample preparation could provide additional environmental improvement.

### 3.8 Comparison with reported methods

The analytical performance and practical characteristics of the proposed EB-based spectrofluorimetric method for FEN determination were compared with representative previously reported chromatographic, spectroscopic, and electrochemical methods (Table S1). The comparison considered analytical performance, sample preparation, analysis time, solvent consumption, applicability, instrumental requirements, and available sustainability assessments. Conventional HPLC methods provide reliable, robust FEN determination and can offer additional advantages, such as simultaneous determination of related compounds and degradation products. However, they generally require chromatographic separation, relatively higher organic-solvent consumption, and more extensive sample preparation. LC-MS/MS methods provide substantially higher sensitivity, with reported detection limits at the ng g^−1^ level, and are particularly advantageous for pharmacokinetic, residue-depletion, and multi-residue investigations. However, these benefits come with high instrumental and operational costs and, in some cases, more complicated extraction procedures. Compared with these chromatographic approaches, the proposed spectrofluorimetric method offers a simpler analytical procedure without chromatographic separation. As summarized in (Table S1), the method provides a linear range of 0.050–3.0 µg mL^−1^ and an LOD of 0.012 µg mL^−1^, together with satisfactory precision (RSD < 2%) and accuracy (recoveries of 98–102%). The complete fluorescence measurement can be performed within ≤4 min, with relatively low instrumental requirements and low solvent consumption. These characteristics make the method particularly attractive for rapid routine determination of the parent FEN. The proposed method also offers a relatively simple sample-preparation procedure. Pharmaceutical samples require minimal pretreatment, whereas plasma samples require protein precipitation with acetonitrile, followed by evaporation and reconstitution. Although acetonitrile use is an environmental limitation of the biological-matrix procedure, the overall method avoids chromatographic mobile phases and extensive chromatographic separation. Therefore, consider the sustainability advantage in relation to the complete analytical workflow rather than solvent use alone. The method was successfully applied to pharmaceutical formulations and spiked plasma and milk samples. In contrast, LC-MS/MS methods reported in the literature are generally better suited for highly sensitive residue analysis and simultaneous determination of FEN metabolites. Thus, the proposed method is not intended to replace high-end chromatographic techniques where ultra-trace detection or metabolite profiling is required; rather, it provides a complementary, rapid, and cost-effective alternative for routine FEN determination and screening under the investigated conditions. We further assessed the sustainability profile of the proposed method using several complementary analytical greenness and sustainability tools, including AGREEprep (0.75), CaFRI (85%), ComplexMoGAPI (83%), BAGI (77.5%), and RGB12 (94.9%). Using multiple assessment approaches provides a broader evaluation of the analytical procedure's environmental and practical characteristics. Nevertheless, these scores should be interpreted together with the limitations associated with acetonitrile use during plasma pretreatment and solvent evaporation.

Overall, the comparison indicates that the proposed EB-based spectrofluorimetric method combines adequate analytical performance with short analysis time, simple operation, low solvent consumption, and reduced instrumental requirements. Its main practical advantage is providing a straightforward approach for FEN determination without chromatographic separation, while complementary mechanistic and computational investigations provide additional insight into the EB–FEN interaction.

## 4 Conclusion

A simple, rapid spectrofluorimetric method was developed to determine FEN based on its fluorescence-quenching interaction with EB. The proposed approach avoids chromatographic separation and provides a practical alternative for FEN determination in pharmaceutical formulations and spiked plasma and milk samples under the investigated conditions.

The combined Stern–Volmer, thermodynamic, Job's, spectral, and quantum-chemical investigations strongly support a predominantly static quenching mechanism involving ground-state EB–FEN complex formation, with electrostatic interactions, hydrogen bonding, and partial charge transfer contributing to the interaction.

Overall, the method combines simplicity, rapidity, satisfactory analytical performance, and reduced instrumental requirements, making it a useful complementary approach to chromatographic methods for routine FEN determination. Further studies using naturally incurred samples are warranted to establish its applicability to real biological and food samples containing FEN residues.

## 5 Limitations

The present study was designed specifically to determine fenbendazole in pharmaceutical formulations and fortified biological samples. The current analytical scope did not include oxfendazole, the major active metabolite of fenbendazole. Accordingly, the potential influence of oxfendazole was not experimentally evaluated and represents a consideration for future studies involving simultaneous determination of fenbendazole and its metabolites.

## Author contributions

A. A. A. M: conceptualized the research idea; supervised the study. A. H. A: writing – review, editing, and supervision. A. A. A: data analysis; manuscript writing, revision, and editing. A. S. T: writing – original draft, methodology, data curation, validation, and investigation. All authors read and approved the final manuscript.

## Conflicts of interest

The authors declare no competing interests.

## Supplementary Material

RA-016-D6RA04629K-s001

## Data Availability

The data and materials used in this study are available on reasonable request. Supplementary information (SI) is available. See DOI: https://doi.org/10.1039/d6ra04629k.
