# Supplementary material for: Eco-friendly spectrofluorimetric determination of fenbendazole in pharmaceutical, plasma, and milk samples using erythrosine B as a fluorescent probe
Source: RSC Adv. 2026 Sep 29. Online ahead of print. doi: 10.1039/d6ra04629k (PMC13621422; doi:10.1039/d6ra04629k)

Supplementary Information (SI) for RSC Advances.
This journal is © The Royal Society of Chemistry

Table S1. Comparative evaluation of the proposed spectrofluorimetric method with previously reported methods for the determination of FEN.

Parameter	HPLC	LC–MS/MS	UV-Spectroscopic	Electrochemical	Proposed spectrofluorimetric method
Linearity range	0.5–10.0 µg/mL	5–1000 ng/g	5–50 µg/mL	0.003–0.599 µg/mL (stirring); 0.003–1.197 µg/mL (static)	0.050–3.0 µg/mL
LOD	>0.1 µg/mL	1.2–1.5 ng/g	—	0.00150 µg/mL	0.012 µg/mL
LOQ	0.5 µg/mL	3.5–4.5 ng/g	—	0.0030 µg/mL	0.043 µg/mL
Sample preparation	Volumetric solution preparation /solvent extraction	Liquid–liquid extraction with protein precipitation and defatting	Binary mixture preparation	Micro-droplet analysis	Simple protein precipitation
Analysis time	10 min	5 min	—	≤4 min	≤4 min
Organic solvent consumption	High	High	—	Low	Low
Application/matrix	Raw materials	Sheep organs and tissues	Laboratory-prepared and commercial binary mixtures	Micro-droplet analysis	Pharmaceutical formulations, plasma, and milk samples
Green assessment	AGREE = 0.76; Eco-scale = 70; BAGI = 85	—	—	—	High green
Instrumental requirements/cost	High	Very high	—	Moderate	Moderate

Figure S1. Comparative spectra illustrating the mathematically summed spectrum, the ET emission spectrum, the experimental spectrum of the ET–FEN mixture, and the FEN emission spectrum.

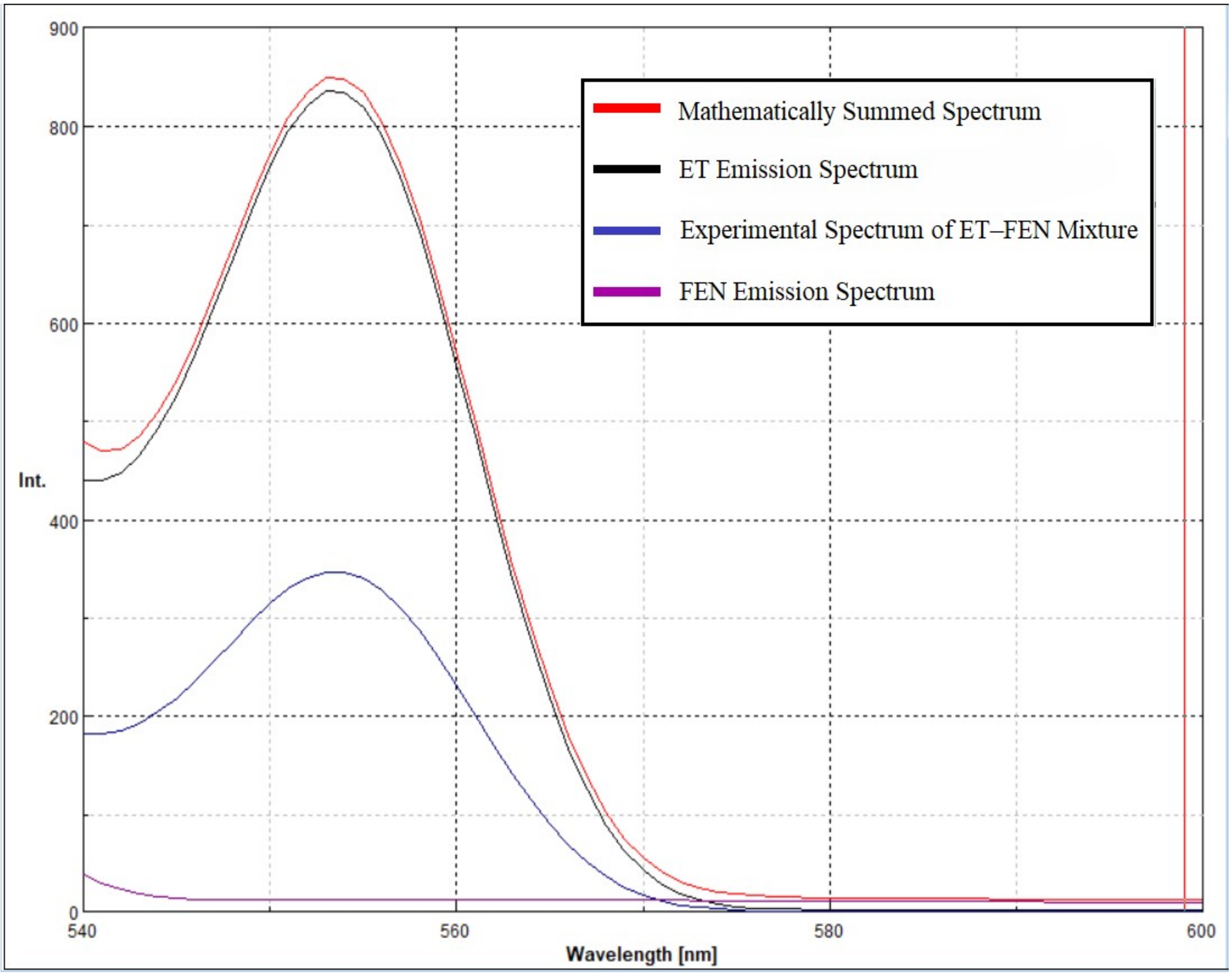

Figure S2. Co-plot showing the UV absorption spectrum of FEN, the excitation spectrum of ET at 527 nm, and the corresponding emission spectrum at 544 nm.

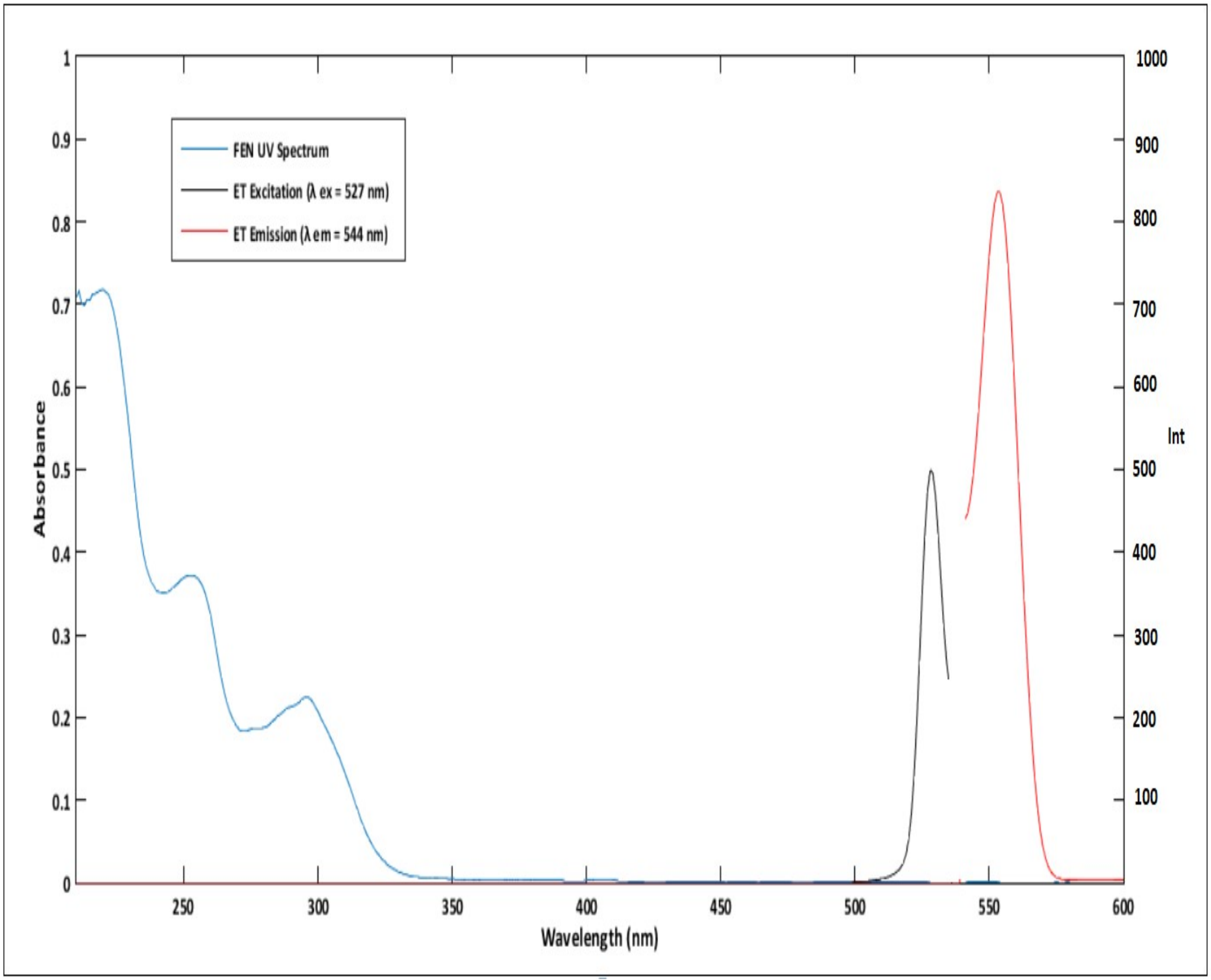

Supplement: Supplementary file 1 [file RA-OLF-D6RA04629K-s001.pdf]
